# Evaluation of paclitaxel-coated balloon angioplasty for the treatment of symptomatic intracranial in-stent restenosis

**DOI:** 10.3389/fneur.2024.1360609

**Published:** 2024-05-22

**Authors:** Huiyuan Xue, Junnan Xi, Xiao Feng Wu, Songsong Feng, Juan Wang, Liwei Chen

**Affiliations:** Department of Neurology, Sanmenxia Hospital of the Yellow River, Sanmenxia, China

**Keywords:** paclitaxel-coated balloon, symptomatic intracranial in-stent restenosis, digital subtraction angiography, cerebral infarction, safety and efficacy

## Abstract

**Background:**

Symptomatic intracranial in-stent restenosis (sISR) poses a major challenge in the management of cerebrovascular diseases, often requiring effective and safe treatment options.

**Objectives:**

This study aims to evaluate the efficacy and safety of paclitaxel-coated balloon (PCB) angioplasty for treating sISR.

**Methods:**

We conducted a retrospective analysis of five patients aged 49-74 years, who were treated with PCB angioplasty between January 2017 and June 2022. Treatment procedures included pre-operative digital subtraction angiography, antiplatelet therapy, and the use of the SeQuent Please balloon. Patients received aspirin and clopidogrel prior to and after the procedure.

**Results:**

The procedure achieved a 100% success rate. The degree of ISR was significantly reduced from an average pre-operative rate of 72±18.9% to a post-operative rate of 34±8.22%. Long-term follow-up showed that the majority of patients did not experience restenosis, confirming the long-term effectiveness of the treatment.

**Conclusions:**

PCB angioplasty demonstrates significant potential as an effective and safe treatment option for patients with sISR, especially those considered to be at high risk. This study supports further investigation into PCB angioplasty as a standard treatment for sISR.

## Introduction

Intracranial atherosclerotic stenosis (ICAS) is a severe cerebrovascular condition. Despite aggressive pharmacotherapy, the annual stroke rate in patients with severe (70–99%) symptomatic ICAS remains alarmingly high, nearly 20% (1, 2). The critical issue with ICAS is its potential to reduce cerebral blood flow, leading to cerebral ischemia or stroke (3–5). Percutaneous transluminal angioplasty with stenting (PTAS) has emerged as an effective remedy for patients unresponsive to pharmacotherapy, reducing stroke recurrence by improving cerebral circulation and perfusion (6–10).

However, in-stent restenosis (ISR) post-PTAS significantly correlates with the risk of stroke recurrence, becoming a major determinant of long-term therapeutic outcomes (11–14). Studies have linked ISR to nearly 80% of postoperative ischemic stroke events (9, 11, 15). As ISR remains a potential limitation for any stenting procedure, the current focus is on restoring targeted blood flow (16).

Recently, drug-coated balloon (DCB) angioplasty, a novel interventional technique, has been recognized as an alternative treatment for ISR (17–19). Both bare and DCB angioplasties have been validated as safe and feasible in treating symptomatic in-stent restenosis (sISR) (18, 20–23). Particularly, paclitaxel-coated balloons (PCBs) are showing promise as a primary method for treating intracranial ISR (24, 25).

Paclitaxel, with its significant antimitotic activity, effectively inhibits cell division and proliferation (17, 19, 26). In cardiovascular interventions, PCBs reduce neointimal hyperplasia by inhibiting the excessive proliferation of vascular smooth muscle cells, effectively preventing or slowing the process of restenosis (17, 19, 27). The application of this innovative treatment tool is particularly significant in the realm of intracranial arterial stenosis management.

Given this context, our study, through a retrospective analysis of five sISR patients, aims to evaluate the efficacy and safety of PCB angioplasty in treating sISR. We explore the real-world clinical performance of PCB, including its impact on ISR, complication rates, and long-term outcomes, hoping to offer a more scientifically valid and effective treatment option for patients with sISR.

## Materials and methods

### Study subjects

**Figure 1:**
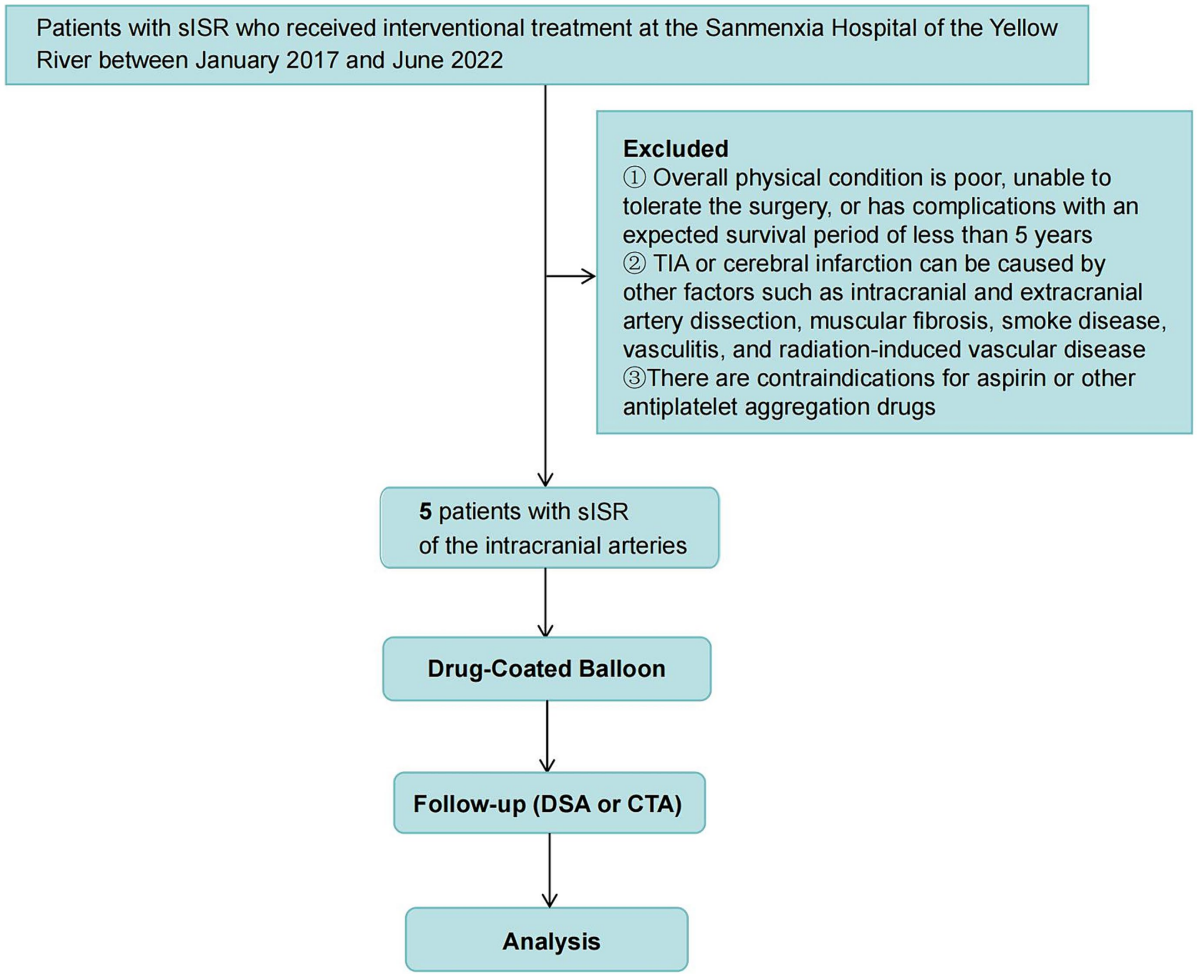
Study flowchart. This figure provides a clear visualization of the study’s methodology and patient selection process.

This retrospective analysis involved five patients with sISR who underwent interventional treatment at Sanmenxia Hospital of the Yellow River between January 2017 and June 2022. The cohort comprised four males and one female, aged between 49 and 74 years, with a mean age of 62.4 years (standard deviation: 9.91 years). Among these patients, two presented with transient ischemic attacks (TIAs), and three had cerebral infarctions (Figure 1). The patient inclusion criteria were as follows: (1) Patients who had undergone PTAS for ICAS, with over 70% ISR and associated TIA or cerebral infarction in the target vascular territory, and who had received standard medical treatment; (2) Patients with a modified Rankin Scale (mRS) score of less than 3. Exclusion criteria included: (1) Poor overall health condition, inability to withstand surgery, or comorbidities with a life expectancy of less than 5 years; (2) TIAs or cerebral infarctions caused by extracranial or intracranial arterial dissection, fibromuscular dysplasia, moyamoya disease, vasculitis, radiation-induced vasculopathy, or other etiologies; (3) Contraindications to aspirin or other antiplatelet aggregation drugs. The study was approved by the Ethics Committee of Sanmenxia Hospital of the Yellow River and followed the principles of the *Declaration of Helsinki*. All participating patients signed informed consent documentation.

### Preoperative preparation

All patients were scheduled for elective surgery. Before the operation, digital subtraction angiography (DSA) evaluations were conducted on all five patients to thoroughly assess the location, degree, and length of the sISR, the diameter of the vessels at both ends of the sISR, the relationship with branching vessels, therapeutic pathways, and collateral circulation compensation. The DSA equipment used was the Allura Xper FD20 system produced by Philips. Patients were administered dual antiplatelet therapy preoperatively, consisting of oral aspirin 100 mg daily (Bayer S.p.A, Import Drug Registration Number: JX20120237, Approval Number: National Medicine Permit Number J20171021) and clopidogrel 75 mg daily (SNC Company, Import Drug Registration Number: H20171237, Approval Number: National Medicine Approval Number J20180029). Additionally, patients received lipid-lowering therapy, opting for either Lipitor 40 mg/day or Crestor 20 mg/day orally, depending on their condition. Antithrombotic treatment continued for at least 5 days before conducting thromboelastography (TEG) to assess the inhibition effect of the antiplatelet drugs. If the aspirin inhibition rate (AA%) or clopidogrel inhibition rate (ADP%) was below 50%, the antithrombotic regimen was adjusted by discontinuing aspirin or clopidogrel and adding oral ticagrelor at 90 mg twice daily. The TEG 5000 Thrombelastograph from Haemonetics Corporation was used for the TEG analysis.

In this study, balloon angioplasty surgeries were performed on five patients with sISR. Each patient underwent the procedure under general anesthesia, receiving an intravenous injection of 3,000 units of heparin to maintain the activated clotting time (ACT) between 250 and 300 s. A right femoral artery approach was used, implanting a 6F femoral artery sheath in conjunction with a 6F MPD guiding catheter. The tip of the catheter was positioned at a straight section of the extracranial part of the affected vessel, which was confirmed through an angiographic review. Using roadmap guidance combined with a micro guidewire, an intermediate guiding catheter or Navien catheter was introduced to a straight section of the vessel proximal to the target lesion. The micro guidewire was maneuvered through the stenotic lesion, placing the wire’s tip at a straight section of the vessel distal to the lesion. A suitable-sized bare balloon (Gateway) was advanced along the micro guidewire, accurately positioned within the stenosis inside the stent, and dilated. Post-dilation, angiography was performed to confirm satisfactory dilation of the stenosis. Then, the bare balloon was withdrawn and replaced with a same-sized SeQuent Please (B. Braun, Berlin, Germany) balloon (PCB). The PCB was slowly dilated again along the micro guidewire into the affected vessel and maintained for 60 s. After removing the PCB, DSA was performed to clarify the degree of residual ISR. Postoperative angiography revealed less than 50% residual stenosis, stent patency, no contrast retention, and good distal vessel display. Finally, the balloon was withdrawn, concluding the surgery.

### Postoperative management

Following surgery, a cranial CT scan was performed on each patient to exclude intracranial hemorrhage. In the absence of bleeding, postoperative dual antiplatelet therapy was continued, comprising oral administration of enteric-coated aspirin (100 mg/d) and clopidogrel (75 mg/d). Additionally, lipid-lowering therapy was prescribed, with options including atorvastatin (40 mg/night) or rosuvastatin (20 mg/night). Postoperative blood pressure was maintained within the range of 100–120/60–80 mmHg (1 mmHg = 0.133 kPa) to prevent hyperperfusion injury. Six months post-surgery, the treatment could transition to monotherapy, with options of either aspirin (100 mg/d) or clopidogrel (75 mg/d). In cases of new-onset neurological symptoms, an emergency cranial MRI or CT scan was advised to ascertain intracranial conditions.

### Follow-up

In this study, all treated patients underwent systematic follow-up at postoperative intervals of 30 days, 3 months, 6 months, 1 year, 2 years, and 3 years. These follow-ups aimed to evaluate the patient’s clinical status and the long-term effectiveness of the treatment. A DSA re-examination was recommended 6 months postoperatively to assess therapeutic outcomes and monitor potential complications. Patients reluctant to undergo DSA were offered alternative imaging methods, such as computed tomography angiography (CTA) or magnetic resonance angiography (MRA). The follow-up period extended up to June 2022, spanning 3 years. Each follow-up session included assessments of vital signs, medication adherence, and adverse events. All clinical incidents were documented by the follow-up physicians and adjudicated by an independent clinical event committee. In the absence of clinical incidents, particular attention was given to ischemic events in the target vessel area within 1 year or to DSA or CTA performed at the one-year follow-up. All angiographic images were sent to an independent core laboratory for review, maintaining blinding to treatment allocation. This review process ensured objectivity and accuracy in treatment assessment.

### Statistical analysis

In this study, all data analyses were conducted using SPSS software version 27.0. Initially, descriptive statistical analysis was performed on the collected data. For continuous variables conforming to a normal distribution, the mean ± standard deviation (x̄ ± s) was used for representation. Categorical data were presented as percentages (%). Appropriate statistical tests were employed to compare differences between different time points or groups; for categorical variables such as surgery success rates or complication rates, chi-square tests or Fisher’s exact tests were utilized. For continuous variables, like changes in stenosis degree, independent sample t-tests or non-parametric Wilcoxon rank-sum tests were applied. Additionally, univariate logistic regression analysis was used to calculate risk factors associated with treatment, such as age, gender, and baseline symptoms, providing odds ratios (ORs) and their 95% confidence intervals (CIs). For primary clinical endpoints, such as ISR or recurrence rates, Cox proportional hazard regression models were employed to analyze hazard ratios (HRs) and 95% CIs. A significance level of *p* < 0.05 (two-tailed) was set for all tests. Moreover, multiple imputations were performed on missing data for sensitivity analysis, ensuring the robustness of the results. This comprehensive and rigorous statistical approach will aid in accurately assessing the effectiveness and safety of PCB angioplasty in treating sISR.

## Results

### Patient baseline characteristics and lesion locations

This study encompassed five patients aged between 49 and 74 years, with an average age of 62.4 years, representing the middle-aged and elderly population. The diversity in lesion locations highlights the complexity of sISR, involving two cases in the V4 segment of the vertebral artery, one in the basilar artery, and two in the intracranial segment of the internal carotid artery. These varied sites potentially influenced the therapeutic approach and postoperative recovery, underscoring the importance of individualized treatment (Table 1).

**Table 1:** Baseline characteristics and lesion location of patients.

Variable	Data
Hypertension [example (%)]	3 (60%)
Type 2 Diabetes	1 (20%)
Hyperlipidemia	2 (40%)
Coronary heart disease	1 (20%)
Non-valvular atrial fibrillation	1 (20%)
Smoking	2 (20%)
NIHSS score before treatment (x ± s)	2.4 ± 1.34
MRS score before treatment (x ± s)	0.8 ± 0.84
Narrowed site	
Vertebral artery V4 segment	2 (40%)
Basilar Artery	1 (20%)
Cervical Internal Carotid Artery	2 (40%)
First-time angiography ISR rate (%, x ± s)	72 ± 18.9
The immediate postoperative ISR rate from the previous surgery (%, x ± s)	24 ± 5.48
Types of stents [example (%)]	
Apoll	3 (60%)
Enterprise	2 (40%)
Time since the last treatment [example[%]]	
≤ 12 months	4 (80%)
> 12 months	1 (20%)
Preoperative ISR rate (%, x ± s)	73.6 ± 4.16
Postoperative ISR rate (%, x ± s)	34 ± 8.22
The incidence of ischemic events within 30 days after surgery [cases (%)]	0 (0%)
Postoperative complication incidence rates [cases (%)]	0 (0%)
Incidence rate of cerebral infarction in the clinical follow-up responsibility vascular zones [cases (%)]	1 (20%)

NIHSS, National Institutes of Health Stroke Scale; MRS, Modified Rankin Scale; ISR, in-stent restenosis.

### Comparative analysis of pre- and post-operative ISR rates

**Figure 2:**
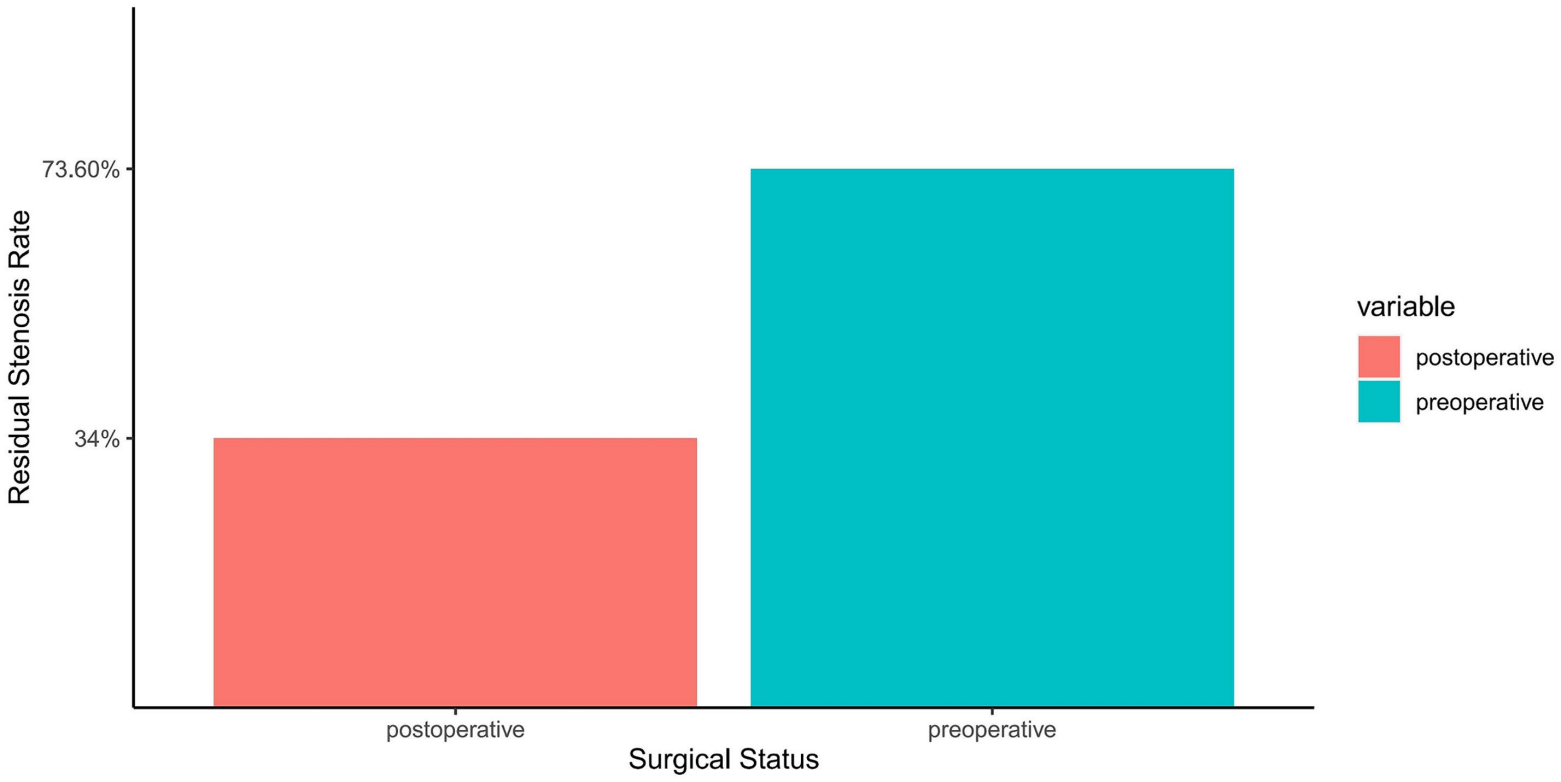
Comparison of ISR rate before and after PCB angioplasty. The horizontal axis represents the surgical status, categorized into preoperative and postoperative groups. The vertical axis shows the percentage of ISR. Two bar graphs display the average preoperative ISR rate (73.6%) and immediate postoperative ISR rate (34%), differentiated by distinct colors for easy comparison. Each bar graph is topped with the corresponding ISR rate, clearly demonstrating the significant improvement resulting from the procedure.

In exploring effective methods for treating symptomatic sISR, PCB angioplasty demonstrated significant therapeutic efficacy. The data revealed that the average preoperative ISR rate of 73.6% significantly decreased to 34% immediately post-surgery (Figure 2). This notable improvement illustrates the effectiveness of PCB angioplasty and may also positively impact the reduction of future cerebrovascular events. The high success rate of this treatment offers a new therapeutic option for high-risk sISR patients and adds an essential component to the comprehensive management strategies for intracranial vascular diseases.

### Surgical outcome and perioperative complication analysis

**Figure 3:**
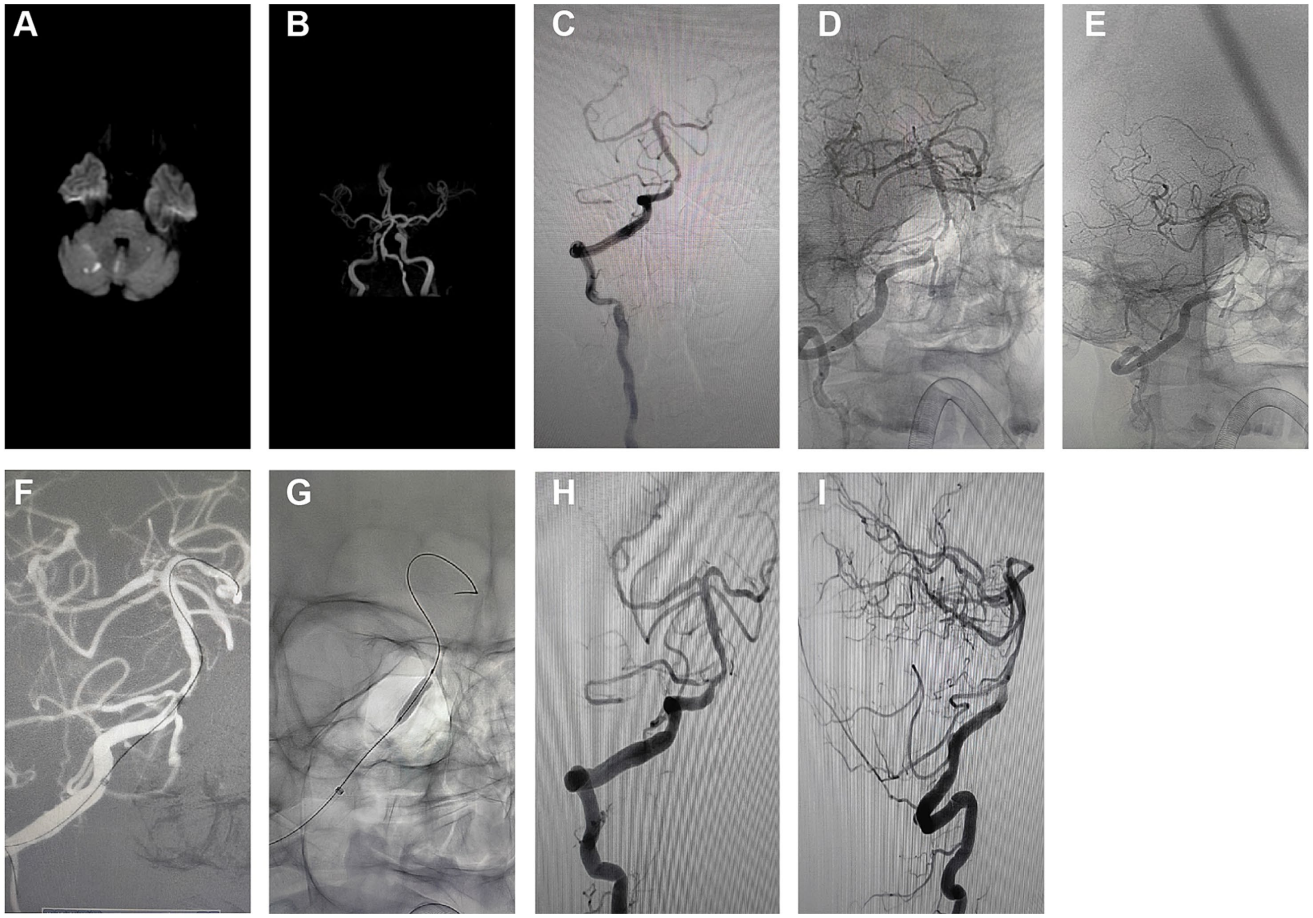
Short-term effects and perioperative safety analysis of PCB angioplasty. **(A)** Initial MRI results indicated multifocal high signals in the cerebellar hemispheres and vermis, suggesting acute infarctions. **(B)** Initial head MRA revealed significant stenosis to near-occlusion in the right vertebral artery V4 segment. **(C)** Pre-intervention angiography showed high-grade stenosis in the right vertebral artery V4 segment. **(D)** Pre-treatment anteroposterior angiographic images were used to evaluate the location of the stenosis. **(E)** Pre-treatment oblique angiographic images further clarified the morphology and extent of the stenosis. **(F)** Angiographic images during balloon expansion depicted the direct radiographic representation of vascular dilation. **(G)** Post-stent implantation angiography exhibited the stent placement and vascular morphology. **(H)** Post-treatment anteroposterior angiographic images demonstrated the restoration of blood flow in the stenosed area. **(I)** Post-treatment lateral angiographic images provided an alternative view of the vascular patency.

This investigation demonstrated a 100% success rate for the procedures, which revealed an immediate and significant reduction in ISR rate post-surgery, indicating the short-term effectiveness of PCB angioplasty. Initially, MRI revealed multifocal acute infarctions in the cerebellar hemispheres and vermis, typical pathological manifestations of vascular occlusion or insufficient blood flow (Figure 3A). Head MRA displayed severe stenosis or near-total occlusion of the right vertebral artery V4 segment, indicating a vascular lesion requiring intervention (Figure 3B).

The initial angiography (Figure 3C) confirmed a 90% stenosis in the right vertebral artery V4 segment, providing direct visual evidence for treatment. Pre-treatment angiographic images in anteroposterior (Figure 3D) and oblique views (Figure 3E) served as baselines for subsequent treatment outcome comparison. These images were crucial for assessing the original state of the vessels and determining the precise location and strategy for interventional treatment.

During the intervention, the first balloon expansion (Figure 3F) recorded the mechanical dilation process of the stenosis site, while the initial Apollo stent implantation (Figure 3G) showed the vascular morphology post-stent placement. These two treatment steps aimed to restore vascular patency and reduce the risk of restenosis.

Post-treatment angiographic images in anteroposterior (Figure 3H) and lateral views (Figure 3I) indicated a significant reduction in ISR rate to below 30%, reflecting the direct outcome of the treatment. The improvement in vascular patency is crucial for preventing future infarction events and can enhance the patient’s quality of life.

In summary, detailed comparative imaging studies concluded that PCB angioplasty is highly effective for improving severe vascular stenosis in the short term. The safety analysis of the perioperative period indicated no significant complications, such as distal plaque embolism, in-stent thrombosis, hemorrhage, or perforator occlusion, suggesting the procedure’s high safety level. This consideration is particularly crucial for patients with intracranial ISR.

### Long-term angiographic monitoring and ISR rate examination

**Figure 4:**
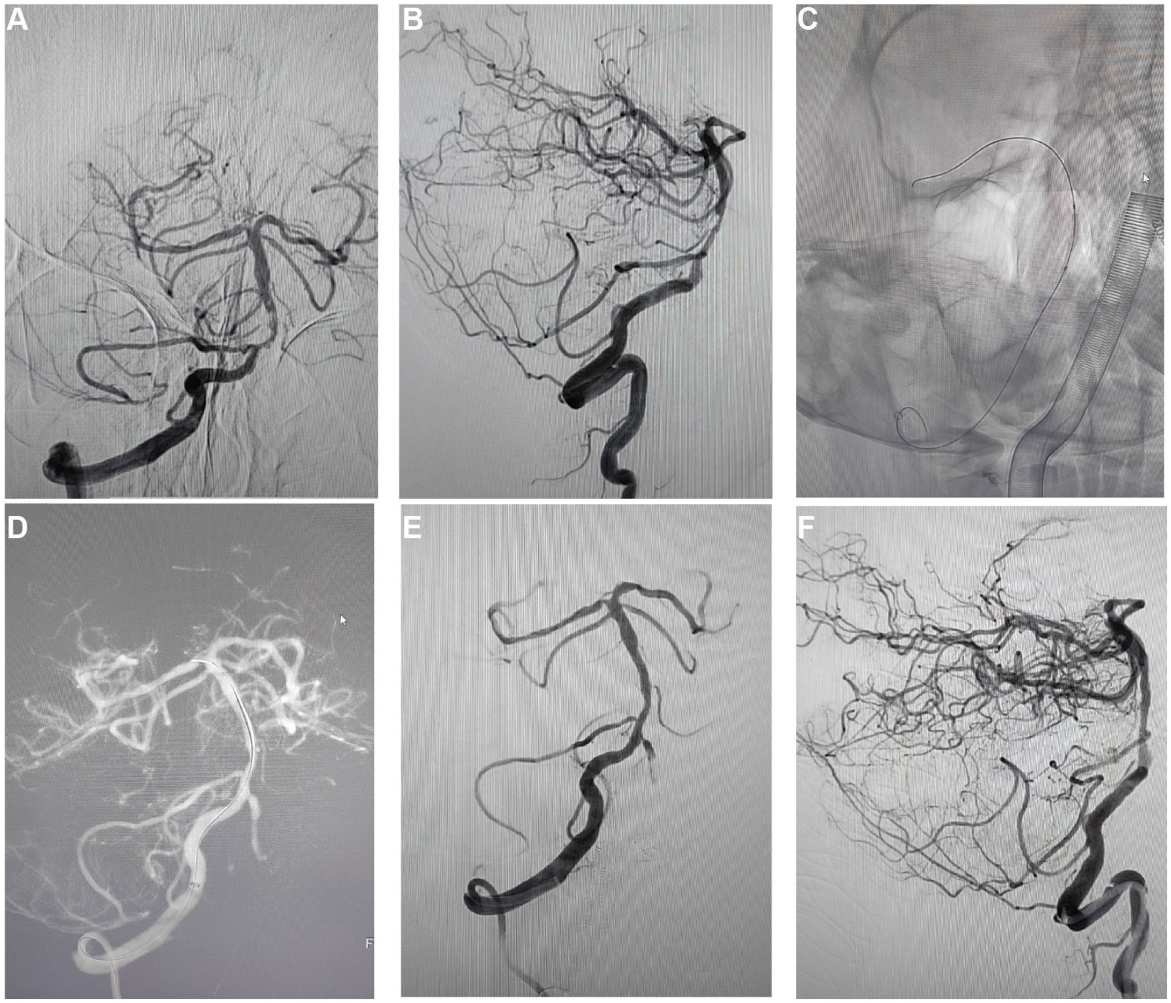
Long-term follow-up and review results after PCB angioplasty. **(A)** A regular 10-month post-operative review, the anteroposterior angiographic images showed restenosis in the treated vascular region, with the degree of stenosis at about 80%. **(B)** Concurrent post-operative lateral angiographic images showed restenosis similar to the anteroposterior view, with stenosis also around 80%. **(C)** Demonstrated the expansion process of the PCB within the previously implanted stent during secondary intervention. **(D)** Angiographic images after balloon expansion recorded the post-dilation vascular morphology. **(E)** Post-secondary intervention anteroposterior angiographic images showed a reduction in the degree of vascular stenosis, with the ISR rate reduced to below 20%. **(F)** Post-secondary intervention lateral angiographic images also showed the ISR rate reduced to below 20%, providing vascular condition observations from different angles.

In the long-term follow-up of patients post-PCB angioplasty, angiographic reviews (Figures 4A–F) assessed the ISR rates. Findings showed that at 10 months post-treatment, some patients experienced an approximately 80% ISR in the treated regions (Figures 4A,B), potentially indicating disease progression or a reduction in initial treatment efficacy. Moreover, 2 out of the 5 patients in the study experienced 80% restenosis within 10 months post-treatment (Table 2). To address restenosis, secondary balloon dilation within the stent was performed (Figure 4C), with post-dilation angiographic images showing improved vascular patency (Figure 4D). Subsequent angiographic images in anteroposterior (Figure 4E) and lateral views (Figure 4F) post-secondary intervention displayed a significant decrease in ISR rate to below 20%, indicating the effectiveness of the secondary intervention in reducing ISR rates and improving vascular patency. These long-term follow-up data provide critical evidence for assessing the lasting efficacy of PCB angioplasty, demonstrating the potential long-term effectiveness of this treatment strategy in the management of sISR.

**Table 2:** Details of the postoperative management and follow-up.

Patient	Additional treatment	Stroke occurrence	Restenonsis
1	Secondary balloon dilation + Medical treatment (Aspirin enteric-coated tablets 0.1 g qd + Rivaroxaban 15 mg qd anticoagulation)	Recurrent stroke during follow-up, treated with tirofiban hydrochloride sodium chloride injection pump for antiplatelet therapy	10 months post-treatment
2	Medical treatment (Standard dual antiplatelet therapy + lipid-lowering)	None	No restenosis
3	Medical treatment (Standard dual antiplatelet therapy + lipid-lowering)	None	No restenosis
4	Secondary balloon dilation + Medical treatment (Standard dual antiplatelet therapy + lipid-lowering)	Recurrent stroke during follow-up, treated with tirofiban hydrochloride sodium chloride injection pump for antiplatelet therapy	10 months post-treatment
5	Medical treatment (Standard dual antiplatelet therapy + lipid-lowering)	None	No restenosis

### Intermediate and long-term follow-up results and complications

**Figure 5:**
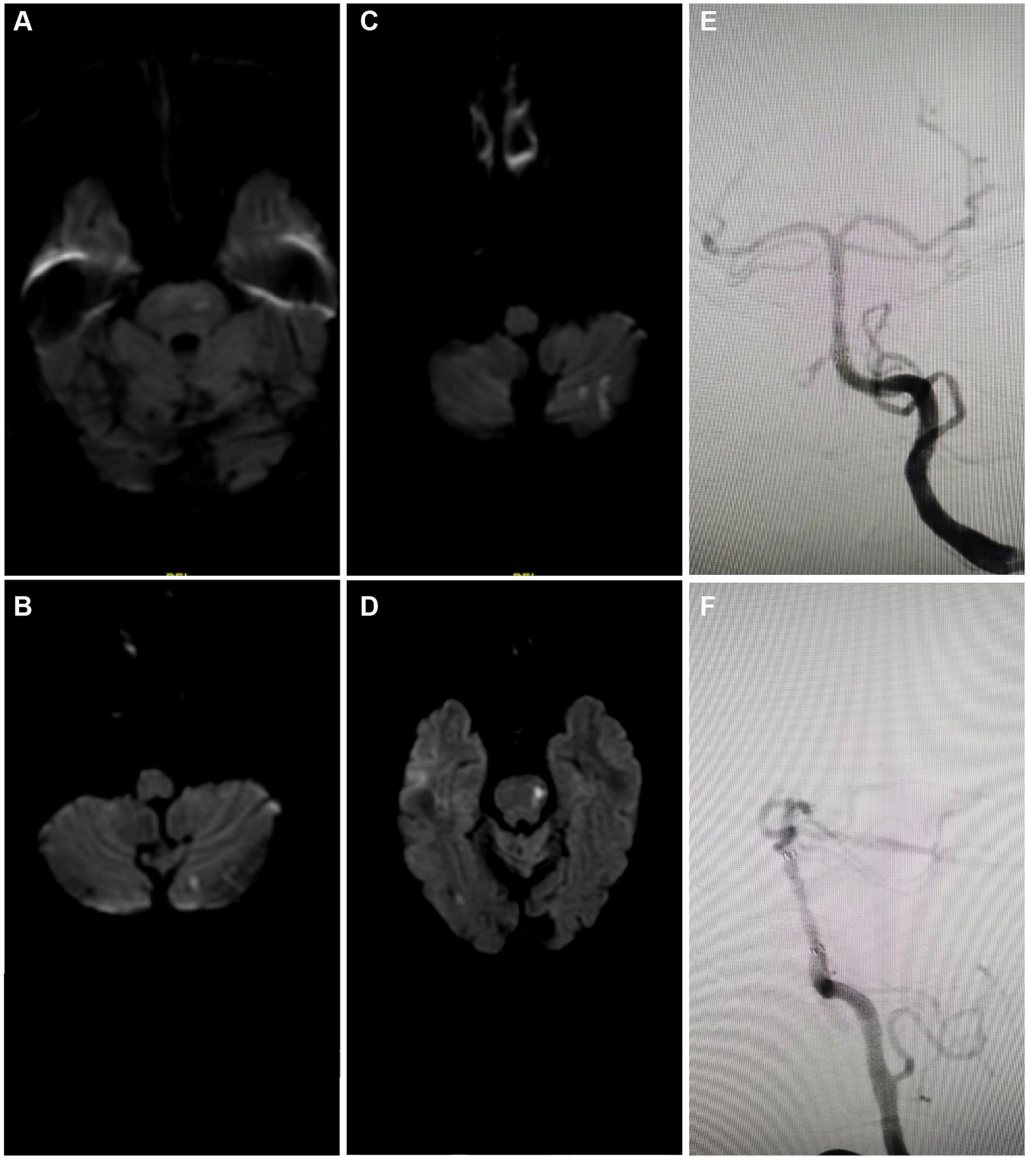
Imaging case analysis of cerebral infarction progression after rivaroxaban treatment. **(A)** New infarct foci in the left pons region 7 months post-treatment. **(B)** New infarct foci in the left cerebellar hemisphere 8 months post-treatment. **(C)** New infarct foci in the left cerebellar hemisphere 17 months post-treatment. **(D)** New infarct foci in the left pons region 24 months post-treatment. **(E,F)** 24-month post-treatment angiographic review following rivaroxaban treatment showed the ISR rate at the lower end of the basilar artery where the stent was implanted was approximately the same as preoperative, about 20%.

The analysis through serial MRI and DSA depicted the progression of cerebral infarctions at different time points post-treatment with rivaroxaban. Diffusion-weighted imaging (DWI) sequences identified new infarcts in the left pons region at 7 months (Figure 5A) and in the left cerebellar hemisphere at 8 months post-treatment (Figure 5B). Further infarcts were observed at 17 months (Figure 5C) and 24 months (Figure 5D) in the same regions. DSA review at 24 months post-treatment (Figures 5E,F) indicated that the ISR rate at the distal end of the basilar artery where the stent was implanted remained at the preoperative level of approximately 20%, suggesting stable blood flow and no significant restenosis at the stent site. Moreover, 4 out of the 5 patients in the study did not experience recurrent ISR during the follow-up period (Table 2). These imaging findings demonstrate that despite the patency of the local vascular stent, new infarcts still occurred at different post-treatment intervals, suggesting that the recurrence of cerebral infarction under conditions of vascular recanalization may be related to other risk factors, such as hemodynamic changes, vascular pathophysiology, or systemic factors. Therefore, comprehensive dynamic monitoring and management of the patient’s overall condition are required in clinical treatment.

## Discussion

Despite the partial effectiveness of traditional methods like pharmacotherapy and bare-metal stent implantation in alleviating symptoms and improving prognosis, the high incidence of sISR remains a significant challenge (22, 28). For instance, bare-metal stents, while temporarily expanding narrowed vessels, fail to effectively prevent neointimal hyperplasia, a leading cause of sISR (29, 30). Moreover, pharmacotherapy, though reducing the risk of thrombosis, has limited effects on already-formed stenoses (31, 32). Therefore, finding a treatment that both effectively dilates narrowed vessels and inhibits neointimal hyperplasia has become a focal point in intracranial artery stenosis therapy (33, 34).

PCBs have emerged as a novel interventional tool in the cardiovascular field (35). Unlike traditional bare balloons or metal stents, PCBs are distinguished by their paclitaxel drug coating on the balloon surface, directly released onto the vessel wall during angioplasty (17, 19, 26). Paclitaxel, an anticancer drug, works by inhibiting microtubule aggregation and disassembly, thereby suppressing cell division and proliferation (36, 37). In cardiovascular treatments, paclitaxel inhibits excessive proliferation of vascular smooth muscle cells, reducing neointimal hyperplasia and effectively preventing or slowing restenosis (38, 39).

This study, through an analysis of five patients treated for sISR with DCBs, achieved a technical success rate of 100%. This finding aligns with previous observations regarding the efficacy of PCBs as documented in prior studies (17, 19, 26). Over a follow-up period ranging from 23 to 42 months, our data indicate that the majority of patients did not experience ISR, further corroborating the potential of PCBs in the treatment of sISR.

Compared to previous research, our findings underscore the advantages of PCBs in preventing vascular restenosis (40–42). Prior to this study, numerous research efforts had already demonstrated significant benefits of DCBs over plain old balloon angioplasty (POBA) in treating ISR. For instance, research by Robert et al. revealed a lower ISR rate following treatment with PCBs for Coronary ISR at 13.0%, compared to a 24.7% ISR rate with uncoated balloons (43). The Third Report of the International DCB Consensus Group on Drug-Coated Balloons for Coronary Artery Disease highlighted that patients treated with PCBs had significantly lower late lumen loss (LLL) and reduced rates of major adverse cardiac events (MACE) and target lesion revascularization (TLR) compared to those treated with POBA (LLL 0.17 ± 0.42 mm vs. 0.38 ± 0.61 mm, *p* = 0.03) (18). Additionally, research by Mahesh Anantha-Narayanan et al. indicated a significant reduction in lumen diameter stenosis percentage (44 ± 33% vs. 65 ± 33%, *p =* 0.01) and binary restenosis (30% vs. 59%, *p =* 0.03) at a 6-month follow-up when comparing DCB to POBA (44). Notably, the immediate post-operative ISR rates and procedure success rates in this study are consistent with early research outcomes in the treatment of cardiac and peripheral vascular diseases. In terms of safety, our study observed no severe complications, aligning with other research reports on the safety of PCBs. However, given the small sample size of this study, further validation in a larger patient cohort is warranted.

Concerning ISR rates, our results’ consistency with literature reports suggests that most sISR events occur within the first year post-stent implantation (45–47). Additionally, our study indicates that different stent types exhibit varying ISR rates (29, 48, 49). Our research also underscores the importance of preventing ISR based on risk factors, such as controlling blood sugar, improving cardiac function, and choosing appropriate stents (10, 15, 50). Moreover, using HR-VWI and CFD analysis can effectively predict the risk of ISR (51).

Currently, there are no unified large-scale randomized controlled trials or authoritative guidelines for the treatment of intracranial ISR (52–55). Nevertheless, PCB angioplasty, as an emerging treatment method, shows promise as a potential option for treating sISR. Our study’s results provide a reliable reference for the clinical use of PCBs, but further prospective research is needed to confirm its precise role in sISR. The findings of this study are promising for clinical practice. Firstly, they offer a new treatment method for sISR, potentially bringing hope to patients unresponsive to traditional treatments. Secondly, given the high-risk nature of intracranial artery stenosis and sISR patients, the use of PCBs provides clinicians with more treatment options. Additionally, since PCBs directly release medication onto the vessel wall, they may reduce patients’ dependence on other drugs, such as antiplatelet agents, thus minimizing medication-related side effects.

While providing essential insights into the treatment of sISR with PCBs, this study also has notable limitations. Firstly, its retrospective analysis nature might introduce selection and information biases, potentially affecting the generalizability and reliability of the results. Secondly, the small sample size limits our ability to interpret and generalize the findings, failing to comprehensively reflect the effects of PCBs across different patient groups. Additionally, the lack of a randomized control group precludes definitive comparisons of PCBs with other treatments, such as bare balloon angioplasty. Lastly, the relatively short observation period precludes a comprehensive assessment of long-term efficacy and safety, particularly regarding chronic neointimal hyperplasia and overall patient quality of life.

Future studies should focus on several areas to address these limitations. Firstly, large-scale, multicenter randomized controlled trials are needed to enhance the reliability and generalizability of findings. Such study designs can more effectively evaluate the relative effects of PCBs compared to other treatments and minimize biases. Secondly, extending the observation period is crucial to assess the long-term efficacy and safety of PCBs, especially regarding their impact on patients’ quality of life. Additionally, further in-depth research into the mechanisms of action and optimal application strategies of PCBs, as well as their effects across different patient groups (e.g., various ages underlying diseases), is essential. Lastly, considering the cost-effectiveness ratio of PCBs, exploring their economic feasibility in clinical treatment is vital to ensure that a broader range of patients can benefit from this treatment modality.

## Conclusion

**Figure 6:**
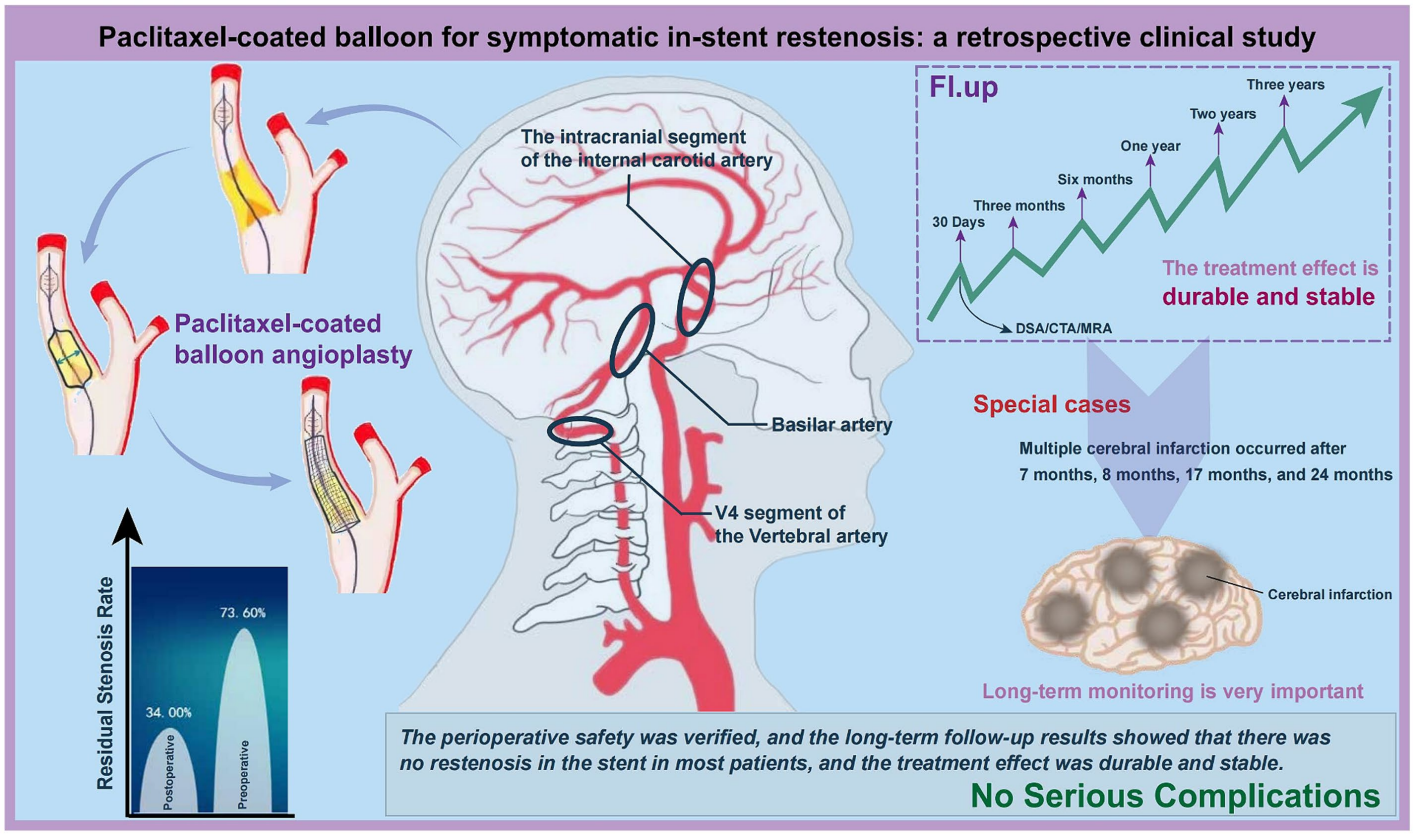
Clinical effectiveness analysis of PCB angioplasty in treating sISR. This figure synthesizes and analyzes the clinical outcomes, highlighting the efficacy of the treatment in managing sISR.

This study, focusing on middle-aged and elderly patients with sISR, underscores the significance of individualized treatment strategies (Figure 6). The average age of patients in this study was 62.4 years, with lesion locations encompassing the V4 segment of the vertebral artery, the basilar artery, and the intracranial segment of the internal carotid artery. PCB angioplasty reduced the immediate postoperative ISR rate, demonstrating significant short-term therapeutic efficacy. The safety during the perioperative period was confirmed, and long-term follow-up indicated that most patients did not experience sISR, showing enduring and stable treatment outcomes. Nonetheless, a unique case involving multiple cerebral infarctions highlighted the necessity for prolonged, close monitoring and the importance of preventive measures. These findings emphasize the potential of PCB angioplasty in treating sISR and the importance of its personalized application.

## Data availability statement

The raw data supporting the conclusions of this article will be made available by the authors, without undue reservation.

## Ethics statement

The studies involving humans were approved by the Ethics Committee of Sanmenxia Hospital of the Yellow River. The studies were conducted in accordance with the local legislation and institutional requirements. Written informed consent for participation in this study was provided by the participants' legal guardians/next of kin. Written informed consent was obtained from the individual(s), and minor(s)' legal guardian/next of kin, for the publication of any potentially identifiable images or data included in this article.

## Author contributions

HX: Writing – original draft, Writing – review & editing. JX: Writing – original draft. XW: Writing – original draft, Writing – review & editing. SF: Writing – original draft, Writing – review & editing. JW: Writing – original draft, Writing – review & editing. LC: Writing – original draft, Writing – review & editing.
